# Participants’ and Health Care Providers’ Insights Regarding a Web-Based and Mobile-Delivered Healthy Eating Program for Disadvantaged People With Type 2 Diabetes: Descriptive Qualitative Study

**DOI:** 10.2196/37429

**Published:** 2023-01-04

**Authors:** Nazgol Karimi, Rachelle Opie, David Crawford, Stella O’Connell, Peter Shane Hamblin, Cheryl Steele, Kylie Ball

**Affiliations:** 1 Institute for Physical Activity and Nutrition School of Exercise and Nutrition Sciences Deakin University Melbourne Australia; 2 Institute for Mental and Physical Health and Clinical Translation Food & Mood Centre Deakin University Geelong Australia; 3 Diabetes & Endocrinology Centre Sunshine Hospital Melbourne Australia; 4 Department of Medicine-Western Precinct University of Melbourne Melbourne Australia; 5 Diabetes Education Services Sunshine Hospital Melbourne Australia

**Keywords:** type 2 diabetes, healthy eating, diet, dietary intervention, low socioeconomic position, digitally delivered, mobile health, mHealth, website, mobile phone, SMS text message, qualitative descriptive

## Abstract

**Background:**

Healthy eating is a key element of type 2 diabetes (T2D) self-management. Digital interventions offer new avenues to reach broad audiences to promote healthy eating behaviors. However, acceptance of these interventions by socioeconomically disadvantaged people (eg, those with lower levels of education and income or from ethnic minority groups) has not yet been fully evaluated.

**Objective:**

This study aimed to investigate the acceptability and usability of EatSmart, a 12-week web-based and mobile-delivered healthy eating behavior change support program, from the perspective of intervention participants living with T2D and health care providers (HCPs) involved in diabetes care.

**Methods:**

This study used a qualitative descriptive design. Overall, 60 disadvantaged adults with T2D, as determined by receipt of either a HealthCare Card or a pension or benefit as the main source of income, were recruited. Data from participants regarding their experiences with and perceptions of the program and longer-term maintenance of any behavior or attitudinal changes were collected through a web-based self-report survey with open-ended questions administered 12 weeks after baseline (54/60, 90%) and semistructured telephone interviews administered 36 weeks after baseline (16/60, 27%). Supplementary semistructured interviews with 6 HCPs involved in diabetes care (endocrinologists, accredited practicing dietitians, and diabetes nurse educators) were also conducted 36 weeks after baseline. These interviews aimed to understand HCPs’ views on successful and unsuccessful elements of EatSmart as a technology-delivered intervention; any concerns or barriers regarding the use of these types of interventions; and feedback from their interactions with patients on the intervention’s content, impact, or observed benefits. All data from the surveys and interviews were pooled and thematically analyzed.

**Results:**

In total, 5 key themes emerged from the data: program impact on food-related behaviors and routines, satisfaction with the program, reasons for low engagement and suggestions for future programs, benefits and challenges of digital interventions, and cultural considerations. Results showed that EatSmart was acceptable to participants and contributed positively to improving food-related behaviors. Most participants (27/43, 63%) mentioned that they enjoyed their experience with EatSmart and expressed high satisfaction with its content and delivery. The educational and motivational content was considered the most useful part of the program. Benefits discussed by intervention participants included gaining health knowledge and skills, positive changes in their food purchasing and cooking, and eating greater quantities and varieties of fruits and vegetables. HCPs also described the intervention as beneficial and persuasive for the target audience and had specific suggestions for future tailoring of such programs.

**Conclusions:**

The findings suggested that this digitally delivered intervention with supportive educational modules and SMS text messages was generally appealing for both participants and HCPs. This intervention medium shows promise and could feasibly be rolled out on a broader scale to augment usual diabetes care.

**International Registered Report Identifier (IRRID):**

RR2-10.2196/19488

## Introduction

### Background

The increasing prevalence of type 2 diabetes (T2D) and the related cost of managing this complicated disease are a major concern worldwide [1]. T2D is particularly concerning among people of low socioeconomic status, who have a higher rate of diabetes and diabetic complications [1]. Self-management is integral in diabetes treatment and requires adherence to several recommended self-care behaviors such as healthy eating, regular exercise, consistent use of medication, and self-monitoring of blood glucose [2,3]. Adherence to self-management activities is challenging, and research suggests that people face a myriad of barriers to doing so, including lack of skills, education, or knowledge [4,5]. Structured diabetes self-management education for people with T2D can improve self-management activities and health-related outcomes, but uptake is low [6]. Previous qualitative studies suggest that some people find face-to-face programs difficult to attend because of a lack of transport facilities, timing of the courses, work or family commitments, or a dislike of group classes [6,7]. The increasing number of people affected by T2D combined with low rates of attendance to structured education programs highlights the importance of developing new and efficient modes of delivering self-management interventions.

Digitally delivered (eg, web- and mobile-based) self-management interventions bypass many of the barriers to face-to-face education and hold a tremendous potential to reach more people and enable them to play an active role in the management of their own health [8]. These interventions offer an opportunity to provide easy access, trusted sources of information, and a low-cost and effective approach for supporting self-care in primary care settings [9-11]. Furthermore, with the rapid development and diffusion of mobile technologies among all demographic groups, these interventions can become widely available to people from different socioeconomic backgrounds [12]. The effectiveness of digital interventions in changing clinical outcomes has been the subject of previous systematic reviews, and their findings have demonstrated improved glycemic control [13,14], diabetes knowledge, self-efficacy, self-care, and exercise behaviors [15].

Although these reports are encouraging, digital interventions have not yet been sufficiently tested in disadvantaged populations, and the mechanisms of action and optimal design of these interventions are not fully understood. There remains insufficient evidence for the acceptability of these interventions among people of lower socioeconomic status, their preferences, and the factors that promote their engagement over time. These aspects need special attention as, if a digital intervention is to be practical, it needs to be accepted, well received, and user-friendly and satisfy the needs of the end user. The acceptability of an intervention can bring about changes in behavior even when medical outcomes show no changes [16,17].

EatSmart, a 12-week evidence-based, theoretically grounded, healthy eating behavior support program, was developed to address this gap. EatSmart was trialed in a group of vulnerable people with T2D. This program involved a simple and practical approach tailored to the needs of people with T2D who face barriers to eating healthily in the context of socioeconomic disadvantage. The feasibility and quantitative impacts of the EatSmart program on participants’ eating behaviors are reported separately (Karimi et al, under review). In summary, feasibility results showed that EatSmart could successfully attract and retain the target group of disadvantaged people with T2D in the first 3 months while the intervention was active. The quantitative results showed that participants demonstrated improvements in vegetable and fruit consumption that were sustained over time (1.22 servings per day increase in total vegetable and fruit intake immediately after the intervention and 1.34 servings per day increase in total vegetable and fruit intake at 6 months after the intervention) as well as improvements in self-efficacy and perceived barriers and enablers to healthy eating after taking part in the intervention.

In this study, using a qualitative descriptive approach, we investigated the perspectives of users and health care providers (HCPs) on this program. This method helped us better understand the nuances of intention, motivation, and triggers to action associated with the program.

### Objectives

The overall objective of this study was to explore the acceptability of the EatSmart program from the perspective of supporting long-term behavior change and to uncover the components of EatSmart that participants and HCPs perceived as most beneficial. For the purpose of this paper, we define acceptability as whether the program was usable and satisfied the needs and requirements of participants.

Specific objectives were to (1) explore intervention participants’ views on their experience with the EatSmart program, including reasons for engagement or nonengagement; (2) explore the longer-term maintenance of any self-reported food-related behavior and attitudinal changes among participants; (3) explore HCPs’ views on successful and unsuccessful elements of EatSmart and their views on concerns or barriers regarding using digitally delivered interventions; and (4) examine HCPs’ feedback from their interactions with participants on the intervention’s content, long-term impact, and any observed benefits.

## Methods

### Design

This study used a qualitative descriptive approach that can provide a comprehensive summarization, in everyday terms, of specific events experienced by individuals [18-20]. This approach allowed for an in-depth understanding of intervention participants’ experiences with the EatSmart program, of HCPs’ views on the EatSmart program in particular, and of digitally delivered interventions in general.

### Intervention

EatSmart, a 12-week, multimodality-delivered healthy eating intervention, included access to a website viewable on mobile devices or PCs and tailored SMS text messages designed for disadvantaged people with T2D. This intervention is described in detail in the protocol paper [21] and summarized in this section. The website consisted of 6 skill-based modules provided to participants on a 2-weekly basis and that covered various topics such as the importance of vegetable, fruit, and whole food intake for health and diabetes; smart shopping planning and food label reading; modification of recipes by trying and incorporating new types of vegetables; improvement of cooking skills and confidence; and a final reinforcement and summary module. The website offered various practical activities such as calculation of current and ideal spending on different food groups, internet-based shopping tours, and food preparation videos. Over the 3-month intervention period, participants also received 3 automated unidirectional SMS text messages per week of various kinds: educational, motivational, and reminders to check the website. The EatSmart intervention involved the delivery of key behavior change techniques such as problem-solving key barriers to healthy eating, self-monitoring consumption, and setting goals for purchasing and consuming key food groups.

### Participants and Recruitment

The EatSmart program recruited 60 socioeconomically disadvantaged people with T2D aged 22 to 75 years, able to read and communicate in English, and with regular access to the internet from 2 outpatient diabetes clinics located at Sunshine Hospital in Victoria, Australia. Individuals who are pregnant or breastfeeding, those with visual or hearing impairment, and those who had an eating disorder with special medical or dietary requirements, clinical depression, planned surgery, or plans for long travel during the study period were not invited to the program. The inclusion and exclusion criteria have been fully described in the study protocol [21].

A total of 90% (54/60) of the participants completed a postintervention survey on the web or by phone with guidance from EatSmart researchers. Those 54 participants were sent an invitation by mail or email containing a plain language statement outlining study details and a consent form to complete a poststudy telephone interview of up to 40 minutes at 36 weeks after the baseline. Of the 54 participants, 20 (37%) consented to interviews, with interviews continuing until data saturation was achieved.

HCPs involved in diabetes care (including endocrinologists, accredited practicing dietitians, and diabetes nurse educators) at Sunshine Hospital diabetes clinics were also invited by email to take part in a short Zoom (Zoom Video Communications) or telephone interview or to complete a web-based survey about the EatSmart program. The same questions were asked in each format (Zoom, phone, and survey). This range of participation options was provided to maximize the number of participants given that HCPs were dealing with the impacts of the COVID-19 outbreaks in Victoria.

### Ethics Approval

Ethics approval was granted by the Western Health Low-Risk Ethics Panel (49763, version 7, dated September 23, 2021) and the Deakin University Human Research Committee (Human Ethics Advisory Groups 186_2019) before conducting this study. All participants were provided with an information sheet explaining the project’s aim and signed a written informed consent form before participating.

### Data Collection From Intervention Participants

Data from intervention participants were collected through a web-based self-reported survey that incorporated open-ended questions to gather qualitative data on feedback on the intervention (the feedback survey), administered 12 weeks after the baseline (time 2), and through semistructured telephone interviews administered at 36 weeks after the baseline (time 3).

#### Self-reported Feedback Survey

The feedback survey included 11 open-ended questions regarding the perceived program effects, useful features of the program, and what the intervention participants liked or disliked about the EatSmart program (Multimedia Appendix 1). Open-ended questions allowed respondents to provide more information and contextual feedback and allowed researchers to better understand the respondents’ true feelings and attitudes about the intervention. Although the questions were primarily qualitative, a small number of categorical and Likert-style response questions were also included to supplement the open-ended questions. These questions were mainly about how useful intervention participants found the SMS text messages and modules.

A personalized link to the web-based feedback survey was sent by SMS text message or email to intervention participants. Telephone assistance from EatSmart researchers was provided to participants who could not complete the survey alone. The data capture and management tool REDCap (Research Electronic Data Capture; Vanderbilt University) [22] was used for data collection.

#### Semistructured Interviews

Semistructured interviews were conducted 6 months after the intervention (36 weeks after the baseline) to provide a more comprehensive description of the acceptability and usability of the program and explore longer-term maintenance of any behavior or attitudinal changes. All interviews were conducted using an interview guide. The interview guide was developed based on the main study research questions and similar studies exploring users’ experiences with digital interventions [16,23-25] and pilot-tested with 2 other researchers, which enabled the interviewer to evaluate the interview guide and ensure that the questions were relevant, clear, and effective for extracting data (Multimedia Appendix 2). The first question invited intervention participants to speak freely and asked the following: “What was your experience with the EatSmart program?” Supplementary questions were asked during the interviews to invite clarification and elaboration. Participants also answered questions about the various parts of the website, SMS text messages, and content presented within the intervention; their perceived capability for action within their personal situation; and adaptation and maintenance of any food-related changes.

The telephone interviews were conducted at a mutually convenient time between October 2019 and November 2019 and were digitally audio recorded with the participants’ permission. The audio recordings were then transcribed verbatim by an independent professional transcription service. Each transcript was subsequently cross-checked with the audio recording for more accuracy and deidentified by NK [26]. Intervention participants received an Aus $20 (~US $13) gift card for their time and participation in the interviews.

### Data Collection From HCPs

Data from HCPs were collected through either a semistructured interview or a web-based survey, with the same questions posed in each format (Textbox 1). Semistructured interviews with HCPs were conducted 36 weeks after the baseline (time 3) at a mutually convenient time for both the HCP and the interviewer. If no mutually convenient time was found, a short survey with open-ended questions was emailed to the HCP. The Zoom web-based platform was used for conducting, recording, and transcribing these interviews, and Qualtrics (Qualtrics International Inc) [27] was used for sending the survey and collecting the responses.

All data from both surveys and interviews with intervention participants and HCPs were pooled to generate a comprehensive picture of postintervention experiences throughout that entire period. These data were then imported into the NVivo software (QSR International) [28] for further analysis.

**Health care provider interview guide.**

**Main and prompting questions**

- Are you aware of any of the patients who joined this program? Did you see any impact of EatSmart on improving healthy eating behaviors or diet-related health outcomes of these patients?
  If yes, can you please explain?
  What do you think could be done to help maintain these healthy behaviors after the program?
  If no, what can be done to make this program more influential for patients who are on low incomes?
- Do you consider EatSmart an effective digital healthy program for patients with type 2 diabetes (T2D) who are on low incomes?
  Yes? What do you think makes a program like this effective?
  No? what do you think makes a program like this ineffective?
- What are the advantages of “digital healthy eating programs like EatSmart” for patients with T2D who are on low incomes?
- What can be the barriers, challenges, or adverse effects of “digital healthy eating programs like EatSmart” for patients with T2D who are on low incomes?
- How do you feel this program might help your own health care or your work as a health professional?
- How do you think this program can be improved for future use on a larger scale?
  How do you think a program like this might need to be tailored to suit patients from different cultural groups that you work with?

### Data Analysis

Descriptive statistics (mean, SD, frequency, range, and percentages) were used to describe the sociodemographic characteristics of the intervention participants and data from categorical and Likert-style questions. Place of birth was dichotomized as “Australian born” and “other,” and education level was grouped as low (year 10 or lower), medium (year 12 or trade, certificate, or apprentice), and high (university degree or higher).

The interview transcripts and open-ended survey responses were combined and thematically analyzed following phases outlined by Braun and Clarke [26]: immersion in the data, data coding to organize the data, creation of major and minor categories, and identification of themes.

The researcher used an inductive approach to derive themes through interpretations of the raw data. To explore various possible interpretations of the data, a random subset of 3 transcripts was independently coded by a second researcher (RSO). Both researchers then met and discussed their interpretations of the data, and the congruence was assessed and found to be good. Open discussions within the research team resolved any discrepancies in data interpretation to reduce researcher bias during the thematic development phase. Researchers agreed on the final category system and accepted it as being representative of the data. To enhance methodological rigor and transparency in the presentation of the methods and results, recommendations of the COREQ (Consolidated Criteria for Reporting Qualitative Research) [29] were followed.

### Researcher Reflexivity

NK, who conducted the interviews, is a dietitian undertaking research in the field of nutrition and physical activity. She was part of the team that developed the EatSmart program; therefore, we acknowledge that there is the potential for social desirability bias from this interviewer. However, steps were taken to reduce social response bias; for instance, (1) at the beginning of each interview, after explaining the reasons for doing this research, it was emphasized to intervention participants and HCPs that the researcher was only interested in their honest thoughts and perspectives on the program and that there were no right or wrong answers, and (2) intervention participants and HCPs were assured that no identifying information (people or organizations) would be published. Intervention participants and HCPs (apart from one) were not known to NK. One of the HCPs (CS) was also a coresearcher on this study.

The other authors were not directly involved in the interviews and analysis. However, they were used as a sounding board on approximately 4 to 6 occasions, where they had the opportunity to encourage further reflection and alternate interpretations of the data (data coding phase and the identification of themes). Owing to ethical considerations, after the interviews, no further contact was made with program participants to obtain more feedback.

## Results

### Participants

Following intervention completion at 12 weeks (time 2), a total of 90% (54/60) of the intervention participants (or, in 7/54, 13% of cases, when participants were not confident in using web-based platforms to complete the survey, carers of intervention participants) completed the feedback survey. The mean age of these participants was 53.6 (SD 12; range 22-75) years; 54% (29/54) of the participants were male individuals; and 41% (22/54) were of the participants Australian born (Table 1). A total of 36 weeks after the baseline (time 3), the data were supplemented with in-depth qualitative phone interviews with 30% (16/54) of the intervention participants. Phone interviews with participants ranged from 25 to 55 minutes in length. Variations in interview length were predominantly because of participant availability or other commitments or contributions of the participants, with some participants providing detailed examples of the changes they had made after the program. After the 13th interview, there were no new themes emerging. Therefore, it was deemed that the data collection had reached a saturation point. Data collection continued for 3 more interviews to confirm and ensure that there were no new themes emerging. The characteristics of the participants who completed the feedback survey at time 2 and those who were interviewed at time 3 are detailed in Table 1. The mean age of participants taking part in the interviews was 49.7 (SD 13.6; range 22-68) years; 69% (11/16) of the participants were female individuals; and 38% (6/16) of the participants were Australian born.

In total, 3 endocrinologists, 2 accredited practicing dietitians, and 1 diabetes nurse educator accepted the invitation to participate in the study as HCPs and share their views through interviews (1/6, 17% of the HCPs) or a web-based survey (5/6, 83% of the HCPs).

**Table 1:** Sociodemographic characteristics of the intervention participants.

Characteristics of the study participants		Time 2 (n=54)	Time 3 (n=16)
Age (years), mean (SD; range)		53.6 (12; 22-75)	49.7 (13.6; 22-68)
**Sex, n (%)**			
	Female	25 (46)	11 (69)
	Male	29 (54)	5 (31)
**Education level, n (%)**			
	High	18 (33)	7 (44)
	Medium	22 (41)	8 (50)
	Low	14 (26)	1 (6)
**Marital status, n (%)**			
	Single	12 (22)	1 (6)
	De facto partnership	6 (11)	3 (19)
	Married	29 (54)	8 (50)
	Divorced	7 (13)	4 (25)
Diabetes duration (years), mean (SD; range)		12 (7.8; 1-30)	11.1 (7.2; 1-20)
**Diabetes medication type, n (%)**			
	Oral diabetes medication	23 (43)	1 (6)
	Insulin	10 (19)	4 (25)
	Insulin and oral diabetes medication	21 (39)	11 (69)
**Place of birth, n (%)**			
	Australia	22 (41)	6 (38)
	Other countries	34 (63)	10 (62)

### Emergent Themes

Five key themes emerged from the qualitative data from intervention participants and HCPs: (1) program impact on food-related behaviors and routines, (2) satisfaction with the EatSmart program, (3) factors contributing to low engagement and suggestions for future programs, (4) benefits and challenges of digital interventions from health professionals’ viewpoint (HCPs only), and (5) cultural considerations (HCPs only). Findings are described in the following sections with corresponding intervention participant and HCP quotes.

#### Theme 1: Program Impact on Food-Related Behaviors and Routines

Participants in the EatSmart program developed various new healthy eating behaviors such as eating, cooking, and purchasing greater quantities and varieties of fruits and vegetables and explained that they maintained these changes 6 months after completing the program.

Most intervention participants (28/54, 52%) highlighted positive changes in their eating behaviors through practical choices. They tried new vegetables or fruits and added more variety to the type of vegetables they ate as side dishes or snacks. Furthermore, they developed new skills such as setting goals for eating a specific number of servings of vegetables per day. Some intervention participants (7/54, 13%) also reported a decrease in consumption of discretionary foods and beverages after the program:

...I minimized or stopped buying processed foods and junk foods and fast foods and stopped drinking soft drinks, even the diet or sugarless soft drinks. Instead, I buy and cook and eat more fruits and vegetables and portion my protein intake and drink more water.

Participant 72, male, aged 50 years, 10 years with diabetes, week 12

Changes in cooking behavior by trying new cooking methods such as steaming or adding more vegetables to dishes were another improvement stated by some intervention participants (12/54, 22%). To a lesser extent, some intervention participants (10/54, 19%) stated that they had created shopping lists and, while grocery shopping, they had visited the fruit and vegetable section first, spent more time there, and bought more fruits and vegetables. Participants also talked about buying frozen and canned fruits or vegetables when fresh items were not in season or were expensive. In addition, there were some changes in the attitudes of EatSmart participants toward their foods and eating behaviors. After the program, intervention participants planned and made more informed decisions about what they wanted to cook and eat. They also became more confident and enthusiastic about trying new types of fruits and vegetables:

...I’m very aware of having some fruit and vegetables every day where previously I would never think about it, if there were vegetables on the plate I’d eat them but I wouldn’t think about, “Oh, I haven’t had any vegetables.” And now there’s fruit in the fruit basket and there’s vegetables in the vegetable crispers...

Participant 2, female, aged 53 years, 12 years with diabetes, week 36

HCPs also perceived the program as effective in improving the food-related behaviors of participants. A total of 50% (3/6) of the HCPs, who had close contact with EatSmart participants, echoed that their participants became more knowledgeable, confident, and determined to adopt new eating habits. They felt that their patients were keen to learn more about the effect of different food items on blood glucose levels and were eager to try new food items.

#### Theme 2: Satisfaction With the EatSmart Program

Informative and motivational content, appealing web design and usability, and acceptable intensity were 3 major categories grouped to create the broad theme of satisfaction with the EatSmart program.

##### Informative and Motivational Content

In terms of informative and motivational content, most intervention participants (29/54, 54%) saw the educational value of the program and stated that EatSmart increased their confidence and motivation to eat healthily:

It increased your knowledge about what to eat and confidence about foods as well...Main text very useful, felt it increased her knowledge. Was very unsure before now, just followed her traditional learning. Now very confident about what to eat, including serving numbers etc...

Participant 78, carer of a woman aged 58 years with diabetes for 14 years, week 12

Intervention participants particularly liked the visual presentation of the educational content on the website and acknowledged that the visual components made the modules more impactful and memorable and, even 6 months after the intervention, they could still remember some of the photos and videos. Participants also said that the visuals motivated them to read the entire module and helped them understand the messages faster.

HCPs also acknowledged that the EatSmart content was appealing. They believed that the content was in line with the needs of people with low income and that the simple language and highly visual messages were the main factors contributing to the success of this program.

##### Recipes

One of the main components of the EatSmart module content that was highly appreciated by many intervention participants and HCPs was recipes. Intervention participants indicated that they liked the recipes and cooking ideas, and some expressed that they saved them for future reference:

...I used to look forward to the recipes that came out with them. Just to give them a go and as I said, something different. Like I’d never had star fruits, whereas now that I’ve tried it, it’s quite nice actually...They were easy, and they were quick...

Participant 7, female, aged 58 years, 6 years with diabetes, week 36

However, a few intervention participants (8/54, 15%) commented that the recipes were either too simple or too complicated for them, which seemed to be related to their existing cooking skills.

HCPs also appreciated the recipes and mentioned that when one is hungry, time-poor, and stretched for money, processed and fast foods can seem like the best choices; hence, introducing easy-to-prepare, budget, and healthy meals in the EatSmart program was useful.

##### Text Messages

During the 3-month intervention, participants received 36 automated SMS text messages. These messages were another satisfactory part of the program for most participants. Intervention participants stated that the educational SMS text messages were very useful and that they felt cared for and were happy to receive them:

...I liked the SMS or phone messages from Stella informing and advising me on the best foods and drinks to eat and how easy it is to prepare and enjoy the varieties and food options available. As a result of being involved with the EatSmart program I’ve lost 10 kilograms weighing from 122 kg to 112 kg today...

Participant 72, male, aged 50 years, 10 years with diabetes, week 12

However, several participants (13/54, 24%) mentioned that fewer SMS text messages per week would be more acceptable.

##### Web Design and Usability

Another major feature that intervention participants found impactful for their satisfaction with the program was the user-friendly design of the website. Many participants (18/54, 33%) appreciated that it was easy to get to the website and easy to follow through and navigate:

it’s pretty straightforward, just scrolling down and clicking to the next page.

Participant 4, female, aged 30 years, 13 years with diabetes, week 36

A total of 50% (3/6) of the health professionals also acknowledged the ease of using the website even for older patients with limited digital skills. One HCP added that the fact that the website required limited data use was also advantageous as disadvantaged participants might not have unlimited data access.

##### Program Intensity

Almost all intervention participants (47/54, 87%) deemed the length of each module to be adequate:

...I found that to be just enough. It was enough for the information to go into my brain and stay there. I think that if you read too much of something you miss a lot. So, I think it was short and concise and to the point that that’s why it sort of stuck with me...

Participant 2, female, aged 56 years, 2 years with diabetes, week 36

HCPs also perceived the short length of the modules to be appropriate and enough to provide essential information. Many intervention participants that were interviewed (13/16, 81%) wanted the program to be extended in terms of the number of modules and duration in future versions. Some suggested that a 6-month program would be ideal, and some believed that weekly modules for a period of 3 months would be better.

##### Overall Satisfaction

Many intervention participants (35/54, 65%) brought about changes in their food-related behaviors and enjoyed the experience with EatSmart, and more than half (27/43, 63%) of the survey respondents reported finding the EatSmart website and SMS text messages very or extremely useful (Table 2).

Some reported that their family members also found the program enjoyable and effective. Intervention participants wanted the program to continue and be expanded to more people with similar conditions. They believed that this program had helped them eat healthier and make the choices that they believed would benefit their diabetes; therefore, all interviewees (16/16, 100%) agreed that they would recommend EatSmart to other people with T2D. All but 1 interviewee, who perceived that they already had enough knowledge, said that they would also continue their engagement with the program if it were available for a longer period.

**Table 2:** Results of the closed-ended questions from the postintervention feedback survey (N=54).

Survey question			Participants, n (%)
**Have you changed the way you buy, cook, or eat food after taking part in the EatSmart program? (n=54)**			
	Do not know	7 (13)	
	No	12 (22)	
	Yes	35 (65)	
**How useful did you find the website? (n=43)**			
	Not at all useful	1 (2)	
	Slightly useful	6 (14)	
	Moderately useful	9 (21)	
	Very useful	6 (14)	
	Extremely useful	21 (49)	
**How useful did you find the phone messages? (n=49)**			
	Not at all useful	1 (2)	
	Slightly useful	4 (8)	
	Moderately useful	12 (24)	
	Very useful	8 (16)	
	Extremely useful	24 (49)	

#### Theme 3: Factors Contributing to Low Engagement and Suggestions for Future Programs

##### Overview

Most intervention participants (27/43, 63%) expressed satisfaction with EatSmart and viewed it as a valuable program. Nevertheless, our data showed that not all of them visited all the modules and read all the SMS text messages (data presented in Karimi et al, under review). The reasons mentioned were grouped into the following four categories: (1) sociocultural factors such as lack of time because of long working hours or family responsibilities; (2) psychological factors such as apathy, lethargy, and perception of inability to use the phone or computer to visit the website; (3) difficulties in accessing the internet during some stages of the intervention; and (4) dissatisfaction with the content.

Concerning dissatisfaction with the content, some intervention participants (7/54, 13%), particularly those who had had T2D for a long period, believed that the program did not offer anything new to them and just reiterated what they already knew. They highlighted the need for further or more advanced information than that provided in the program. Although some of these participants felt that there was little new information, they nevertheless considered that EatSmart was helpful as it provided healthier food options or reinforced their previous knowledge. In addition, 4% (2/54) of the participants perceived that the program content contradicted their previous nutritional beliefs or their culture; therefore, they were reluctant to read or follow the recommendations:

...some things contradicted her thoughts about diabetes. Thought the website (was) associated with western medicine and doctors, so this put her off reading more...

Participant 82, carer of a woman aged 44 years with diabetes for 14 years, week 12

##### Suggestions for Future Programs

Several suggestions were made on how to enhance the program. Intervention participants believed that the program could be improved with greater tailoring to individual needs; more advanced diabetes-specific information; more recipes; and more features, such as a web-based discussion board.

In the design of EatSmart, all participants received the same set of basic and essential nutrition-related skills and information necessary for managing T2D. This was done to maximize the usability of the program for participants with very little knowledge of the relationship between food and diabetes. However, some participants (10/54, 19%) noted that people at different stages of diabetes have different needs in establishing or changing eating behaviors, and they looked for information that was relevant to the complications that they faced. Therefore, they requested more detailed diabetes-specific information such as the carbohydrate content of different fruits and vegetables, best drink choices, and low-carbohydrate diets. A participant suggested that future programs would be more beneficial if they included some dietary advice for people who struggle with different conditions at the same time, for example, diabetes and arthritis.

Furthermore, intervention participants described the desire to have recipes that accommodated varying characteristics and circumstances, such as different cooking skill levels, food preferences (eg, vegetarianism), and families’ food preferences. They desired more traditional recipes or techniques to incorporate more vegetables into traditional foods from particular cultures. The inclusion of low-carbohydrate, “quick and easy” recipes that are suitable for working women was another suggestion made for future programs:

...I liked the recipes there; they were simple and easy to prepare. But maybe add more traditional recipes (Indian foods) or show how we can improve our traditional foods and make them healthier by adding vegetables...

Participant 13, male, aged 68 years, 20 years with diabetes, week 36

Although participants highlighted the benefits of an automatic web-based program, a complementary theme was identified in which the importance of support from others (HCPs, peers with lived experience, or other people with similar conditions) was expressed. Several participants who were interviewed (8/16, 50%) suggested adding a web-based forum or discussion board where they could share their problems or experiences with other participants or the research team.

HCPs also recommended some improvements in content to increase the efficacy of EatSmart or future similar programs. They specified the need to discuss the common food- and budget-related problems of disadvantaged people with diabetes on the website. One HCP advised the addition of content on how having a healthy diet can help patients save money by preventing diabetes complications and their related medical expenses.

Similar to the participants, HCPs also spoke of the importance of introducing more affordable recipes using commonly available nutritious foods:

...Use examples of foods that are readily available and affordable recipes of popular foods prepared in a healthy way. Information regarding carbohydrates in popular “unhealthy” food, when they may be able to have them and how much exercise needed to offset it Regular updates on new recipes etc...

HCP 5, endocrinologist, week 36

#### Theme 4: Benefits and Challenges of Digital Interventions From HCPs’ Viewpoint

The digital environment was seen by all the HCPs (6/6, 100%) as a promising platform to deliver support and information needed by people with diabetes. They acknowledged that many of their patients, especially the younger ones, were already engaged with digital technologies, and some were interested in receiving digitally delivered health programs. HCPs believed that digital interventions could provide an important adjunct to their routine medical care, facilitate the delivery of support and education, and save consultation time:

...Well, I think, having that program to refer people to, it’s something they can take home and do after because often in a consultation you don’t have time to go through everything that you’d like to cover, but if you can cover some essentials in the consultation and then give that program to the patient to say look, you can take time at your convenience go through at your own pace, the different parts of the program and then, if you’ve got questions at our next consultation we could discuss those and reinforce anything that needed to be reinforced...

HCP 6, credentialed diabetes educator, week 36

HCPs also emphasized that this mode of program is affordable, “free,” and easily accessible and, in addition to making the right information available at the right time and in the correct form, can help patients save on travel time.

Although digital interventions were acknowledged to simplify the transmission of essential information to patients, some HCPs (2/6, 33%) articulated concerns about the reliability of the materials and stressed the importance of providing up-to-date evidence-based information. In total, 50% (3/6) of the HCPs also expressed unease about information being misinterpreted by patients, which may cause harm to them. One HCP emphasized that these programs needed to be introduced only by HCPs and as an adjunct to their care after the first visit:

Another adverse effect can be a misunderstanding about the serving sizes. You tell them to eat two serves of fruits and five serves of vegetables, but they may think the more the better so overeat different fruits. Because of this, I suggest the introduction of this program should be after the first session with a dietitian. They need to learn the basics first, then step by step learn new information.

HCP 2, accredited practicing dietitian, week 36

Another concern of HCPs was the possibility that participants might overlook the role of the health care team after participating in these programs. A total of 50% (3/6) of the health professionals talked about the perceived detrimental effects on the health professional-patient relationship with the advent of health-related digital technologies and shifting the control of information from the health care team to digital programs. HCPs argued that, regardless of advances in digital interventions, these programs provide general information rather than customized recommendations for the special needs of patients; thus, the need for communication between health professionals and patients should not be neglected.

#### Theme 5: Cultural Considerations

HCPs highlighted that food practices can be affected by cultural identity and, therefore, it is important to consider cultural differences in both the intervention content and design.

In terms of content, HCPs suggested the addition of more diverse food items and recipes to the website that cater to different ethnic and cultural backgrounds. One of the health professionals emphasized that participants from different ethnic backgrounds are probably familiar with how to cook their traditional foods. Hence, it is valuable that, instead of merely providing the recipes, the program shows them how to make their traditional foods healthier by, for example, adding more affordable vegetables. Another HCP also suggested covering different food beliefs such as vegetarianism along with cultural considerations:

...It would be good to have some that specified the different cultural groups or different eating beliefs like even one for vegans one for vegetarians that might not even be about their particular ethnic culture, but it could be just about their beliefs around food...

HCP 6, credentialed diabetes educator, week 36

Regarding technical design, all HCPs suggested translating the website to languages other than English so that it can be usable by non-English speakers. In addition, HCPs suggested using culturally familiar elements for specific ethnic groups, for instance, incorporating pictures and videos of people of their own nationalities into the design of websites.

## Discussion

### Principal Findings

The findings showed that a digitally delivered intervention with supportive and educational modules and SMS text messages improved healthy eating and was accepted by people with diabetes who were socioeconomically disadvantaged. The study also found that HCPs generally held positive views of digital programs in promoting healthy eating behaviors and had specific suggestions for tailoring such programs, including to cultural groups.

#### Changes in Food-Related Behavior

EatSmart targeted nutritional knowledge as well as promoting positive mindsets toward healthy eating (ie, self-efficacy). Participants reported an increase in their nutritional knowledge and confidence in improving their eating behaviors. Moreover, many perceived that the program had led them to eat more vegetables and fruits. These changes were confirmed by HCPs who had direct contact with the participants.

These changes may have been brought about by the intervention material, increasing participants’ intentions and forethought about improving their eating behaviors. Bandura [30] proposed that providing information about a behavior or cues to perform a behavior is important for producing intention to change. This is regulated by forethought, in which people guide their actions by considering anticipated future behaviors and their effects. Over time, forethought contributes to increase self-efficacy [30].

Similar results were reported by Arora et al [31], Moussa et al [32], Porter et al [33], and Ruggiero et al [34], who explored the effects of different kinds of digital interventions on food-related behaviors of disadvantaged people with T2D. These studies found positive intervention effects on nutritional knowledge, diabetes knowledge, self-efficacy, and nutritional behavior.

#### Program Intensity

Many EatSmart participants (32/54, 59%) perceived the frequency of the modules to be appropriate, although a few (13/54, 24%) were dissatisfied with the number of SMS text messages per week. Previous evidence suggests that more frequent contact from an intervention is associated with greater effects on behavior change, whereas, by contrast, SMS text messages sent too frequently can elicit negative reactions and result in disengagement or adverse outcomes [25,35]. For example, Horner et al [35] found that intervention participants with T2D who received physical activity–related SMS text messages twice daily for 6 months developed negative feelings about the program and started avoiding or ignoring messages. That study and others [25,36,37] found that participants questioned the intrusiveness of receiving frequent SMS text messages. It remains unclear how digital interventions can deliver accurate and timely information at a sufficient intensity without unfavorable effects on participants’ engagement. Some studies have indicated that patient preferences for the number of SMS text messages may depend on the message type. A large and varied SMS text message database can improve participant engagement as opposed to frequent messages with similar content, which can cause boredom or indifference [25,38]. Updating delivery frequency according to participant preferences after regular check-ins may also help negate the negative feelings that result from frequent SMS text messages [39].

#### Engagement With the EatSmart Program and Directions for Future Programs

Participants reported good engagement with different components of EatSmart in general, which attests to the overall acceptability of the program. However, almost half (23/43, 53%) of the participants reported not fully engaging with all components. Some participants (7/54, 13%) suggested that they would have preferred more detailed diabetes-specific information or more customized information as the basic content and familiarity with the content were the main reasons for their lower engagement. Many previous studies [6,40-45] have discussed patients’ perceptions of having sufficient knowledge as main reasons for lower engagement or nonattendance to diabetes education programs. Horigan et al [6] have suggested that many people are simply rejecting educational opportunities without fully understanding what is involved and what they might gain from attending and that, if all aspects of diabetes education were explained to participants, it might increase engagement [6]. In a study by Temple and Epp [42], when noncompleters were given more information about the intervention, many said that they would be willing to complete or expressed a wish to learn more.

In this study, lower engagement and suggestions for more advanced content mainly came from participants with a longer diabetes duration. Similarly, other studies have shown that program satisfaction and uptake were lower among people who had a longer diabetes duration, with newly diagnosed participants more likely than others to complete the course [7,46]. This suggests that, although diabetes self-management education and support should be offered to everyone with T2D, people who are newly diagnosed should be specifically targeted in the first year following diagnosis, and more advanced and specifically tailored programs should be designed for people with a longer diabetes duration.

A 2017 review of diabetes education programs [6] stated that there are a multitude of other reasons why people with diabetes may have lower engagement with digital interventions, including the nonprioritization of health interventions, lack of enthusiasm, and the belief that they would not benefit, contributing to poor uptake and completion. Our study results also corroborate these findings.

In the EatSmart program, both intervention participants and HCPs expressed high satisfaction with the recipes and videos of food preparation within the intervention, and one of the suggestions made to improve the program was adding more similar materials to the website, such as more recipes incorporating affordable foods. This finding highlights the importance of direct instruction and observational learning in interventions with this target group. Observational learning involves people observing a behavior conducted by others and then replicating those actions. This is often exhibited through “modelling” of behaviors. Modeling provides individuals with the skills and strategies to adopt and maintain behaviors [47]. The positive effect of modeling on improving diabetes self-management among people from different sociocultural backgrounds has been reported in previous studies [32,34,48-51].

Intervention participants also suggested adding more interactive features such as a web-based forum or a discussion board to provide opportunities to interact with other people with diabetes or HCPs in a way that is anonymous and convenient. This demonstrates the importance of delivering social support, which is an effective technique for behavior change. The facilitation of social support in electronic health programs has been shown to increase health outcomes above and beyond programs without this feature (Hales et al [52]). Evidence has demonstrated that being a member of a web-based diabetes forum resulted in better engagement with programs and improved glucose control, reduced HbA1c levels, and improved dietary choices and led to a better understanding and increased confidence in managing diabetes [6,34,53]. However, a qualitative study showed negative attitudes toward peer support in health promotion programs [54]. The authors of this UK study expressed the need to further investigate how to foster engaging and enjoyable social support environments in interventions [54]. Further important considerations regarding web-based forums are the additional ongoing cost and resources required to host the forums and ensure their safety, maintain the currency and relevancy of the information presented there, and moderate the discussion between participants [55]. Previous research has also indicated that providing a high level of support from HCPs is more important for people of low socioeconomic status, who often have lower health literacy, material resources, and self-efficacy to cope with the complex burden of self-care [54].

#### HCPs’ Perspectives on the Benefits and Challenges of Digital Interventions

Our findings showed that EatSmart was well received among HCPs as it was felt to be both feasible and sustainable to support their patients from a workload standpoint. Consistent with previous studies [55-58], HCPs in this study stated that the use of digital platforms is a promising method to improve patient agency and empowerment by providing disease-related knowledge and education, provided that concerns about patient-HCP contact and reliability of information were addressed. HCPs also acknowledged that, rather than relying on occasional in-clinic interactions, digital interventions have the potential to benefit health care by overcoming constraints because of limited clinician time and inability to provide meaningful interventions at the most appropriate time. Furthermore, in line with previous literature [13,53,59-61], they mentioned that digital interventions can broaden the availability of services through ease of access, reducing health care inequities and increasing cost-effectiveness through prevention and self-management.

In this study, HCPs expressed some concern about the possible negative effects of digital interventions on routine clinical care and patient-HCP interactions. However, the current literature suggests that such educational and behavior change platforms hold value to patients by facilitating improvements in communication with HCPs and enabling a level of understanding that supports shared decision-making in clinics [62]. Furthermore, appointment reminders can be added to these interventions to encourage session attendance with HCPs. Another element of concern for HCPs was related to the reliability and appropriateness of the content of digital interventions, which may limit their use in clinical care. This concern has been similarly expressed in previous studies [55], which noted that poorly designed health and medical apps have the potential to harm the users. Gurupur et al [63] discussed that many readily available low-cost health apps are not based on evidence from research and that such apps may provide incorrect information. To address this concern, efforts need to be made to ensure that digitally delivered diabetes programs are evidence based, theory driven, quality assured, and regularly audited for necessary updates to content.

#### Cultural Considerations

EatSmart shows promise in delivering the essential knowledge and skills necessary to eat healthily. However, engaging with this program can still be challenging for many ethnic minority groups because of socioeconomic, linguistic, and cultural barriers. HCPs in this study emphasized the critical importance of tailoring the content and design of digital interventions to fit the cultural identity of patients. Previous studies and meta-analyses of technology-based interventions [13,25,33-35,64-67] have emphasized the importance of customizing the interventions in a way that meets the cultural and linguistic needs of the target population. However, studies on the effect of interventions that address these needs have produced mixed results. For instance, Arora et al [31] found improvements in eating habits and medication adherence but not in glycemic control; Moussa et al [32] found improvements in knowledge, self-care behaviors, and glycemic control; and Wayne et al [68] found improvements in glycemic control but not in self-care behaviors.

Tailoring program content to culture or language may be more complicated, costly, and time-intensive to implement but may make it more persuasive, salient, and useful. Further research should be conducted on how to execute precise tailoring and customization on a cost-effective basis and what types of personalization will result in more persuasive cues.

### Strengths and Limitations

The strengths of this study included the qualitative approach, which enabled us to gather rich data about participants’ experiences with the EatSmart program and HCPs’ insights on successful or unsuccessful elements of EatSmart. These data were based on responses from 54 participants of different ages, education, occupations, living arrangements, and marital statuses, which is a relatively large and demographically varied sample for a qualitative study. The complementary one-on-one interviews with participants and HCPs allowed for triangulation of the data to gather richer descriptive insights. Exploring the perspectives of both patients and HCPs was a further strength. Other strengths included the application of Social Cognitive Theory to both the intervention design and the consideration of the results, which allowed us to make a theoretically informed interpretation of the qualitative data.

Despite the study strengths, our results should be interpreted with consideration of the following limitations. First, the findings regarding HCPs’ perspectives are limited by their small number. This may have limited the variability in responses and precluded comparing results across different HCPs or disciplinary backgrounds. However, all the health professionals involved (6/6, 100%) had extensive experience in providing services to vulnerable patients and were aware of the special needs of these groups. Second, this study was conducted in English, limiting some participants (with English as their second language) regarding precisely expressing their opinions and elaborating on their ideas. It was also conducted in a metropolitan area and so generalizability to those in rural or remote areas is unknown. Moreover, another possible limitation to the generalizability of results might be the limited access to digital technologies by people experiencing severe socioeconomic disadvantage, such as those who are homeless. However, Australia has a high diffusion of mobile technologies among all demographic groups, and the digital divide is shrinking steadfastly, with currently nearly 9 out of 10 adults owning a smartphone [69]. A further limitation was our inability (because of the ethical limitations) to invite people who were offered the program but did not register to take part in it or those who withdrew from the program to take part in interviews. Interviewing these people could have helped us better understand why some people are not willing to use phone- or web-based structured programs or were unsatisfied with the program. Finally, people with T2D and HCPs who chose to take part in the study interviews may have been a particularly motivated group who had a positive experience with EatSmart.

### Conclusions

The findings indicate that EatSmart, a behaviorally focused nutrition intervention, was generally appealing to socioeconomically disadvantaged people with T2D and showed promise in terms of promoting healthy eating behaviors and increases in vegetable and fruit consumption that were sustained over time. HCPs also found this intervention beneficial and persuasive for the target audience.

We identified specific objective elements of the intervention, such as informative content, appealing design, and acceptable intensity of the program. The web and text content was found to be engaging and provided an appropriate source of knowledge and psychosocial support for people with diabetes. On the basis of Social Cognitive Theory, we conceptualized that knowledge increased the self-efficacy of participants and was used by them to make and maintain changes in food-related behaviors. Improvements for future studies were identified, including tailoring to the frequency of contact preferences, existing knowledge, and cultural background, as well as enhanced opportunities for social support and verbal communication.

If specific adaptations are made, the intervention could feasibly be rolled out on a broader scale as an inexpensive adjunct to routine clinical care to promote healthy eating and diabetes self-management among vulnerable populations. More work is required to make digital interventions more appropriate for people from different cultural backgrounds. Future studies are required to investigate whether increased customization results in increased engagement of more disadvantaged participants and improved health outcomes.

## Appendix

Multimedia Appendix 1 Postintervention feedback survey.

Multimedia Appendix 2 Interview questions for intervention participants.

## Abbreviations

COREQ: Consolidated Criteria for Reporting Qualitative Research
HCP: health care provider
REDCap: Research Electronic Data Capture
T2D: type 2 diabetes
