# Supplementary material for: Participants’ and Health Care Providers’ Insights Regarding a Web-Based and Mobile-Delivered Healthy Eating Program for Disadvantaged People With Type 2 Diabetes: Descriptive Qualitative Study
Source: JMIR Form Res. 2023 Jan 4;7:e37429. doi: 10.2196/37429 (PMC9893734; doi:10.2196/37429)
Supplement: Multimedia Appendix 1 [file formative_v7i1e37429_app1.docx]

**Multimedia Appendix 1**

Post-intervention feedback survey

| **Questions** | **Response options** |
| --- | --- |
|  |  |
| Over the past 12 weeks, did you look at the Eat Smart website? If yes selected: How useful did you find the website? | 0= Yes, 1= No, 3= Don’t know  1= Not at all useful, 5= Extremely useful |
| What were the MOST useful parts of the website? | Descriptive text |
| What were the LEAST useful parts of the website? | Descriptive text |
| What was one main message you remember from looking at the website? | Descriptive text |
| In the past 12 weeks, did you receive phone (SMS text) messages from us on your mobile phone? If YES selected: How useful did you find the phone messages? | 0= Yes, 1= No, 3= Don’t know  1= Not at all useful, 5= Extremely useful |
| How many of the phone messages did you read? If you didn't read them all, can you please let us know why? | 0= All, 1= About three-quarters, 2= About half, 3= About a quarter, 4= None  Descriptive text |
| What were the MOST useful parts of the phone messages? | Descriptive text |
| What were the LEAST useful parts of the phone messages? | Descriptive text |
| What was one main message from the phone messages that you remember? | Descriptive text |
| Have you changed the way you buy, cook or eat food after taking part in the Eat Smart study? If YES, can you please explain how you have changed the way you buy, cook or eat food? | 0= Yes, 1= No, 3= Don’t know  Descriptive text |
| Overall, what did you like most about being involved in the Eat Smart study and why? | Descriptive text |
| Do you have any suggestions for how we could improve this study? | Descriptive text |
