# Supplementary material for: Participants’ and Health Care Providers’ Insights Regarding a Web-Based and Mobile-Delivered Healthy Eating Program for Disadvantaged People With Type 2 Diabetes: Descriptive Qualitative Study
Source: JMIR Form Res. 2023 Jan 4;7:e37429. doi: 10.2196/37429 (PMC9893734; doi:10.2196/37429)
Supplement: Multimedia Appendix 2 [file formative_v7i1e37429_app2.docx]

**Multimedia Appendix 2**

Interview questions from intervention participants

**Western Health Low Risk Human Research Ethics Panel**

**Interview Guide for Patients**

**Version:** 2 **Dated:** 10.09.2020
**Site:** Sunshine Hospital

**Full Project Title:** A novel approach for supporting healthy eating on a budget for people with Type 2 diabetes

**Principal Researcher:** Professor Kylie Ball

**Co-principal investigator:** Associate Professor Peter (Shane) Hamblin

**Associate Researcher(s):** Dr Rachelle Opie, Professor David Crawford, Ms Nazgol Karimi, Ms Cheryl Steele

Hello, MR./Mrs./Ms….

My name is Nazgol Karimi. I am a PhD student from Deakin University Institute of Physical Activity and Nutrition.

I am calling to have a phone interview with you about EatSmart, a healthy eating on a budget program you had joined before.

EatSmart was aimed to provide practical knowledge and skills for people living with Type 2 Diabetes to improve their healthy eating behaviours as a key component of diabetes self-management.

You were an important member of that program and the purpose of this interview is to hear your views and experiences of EatSmart, particularly now, 6 months after you took part.

Your participation in this interview is completely voluntary. This means that you do not have to participate in this interview unless you want to. And if you agree to participate you have the right to only answer the questions you choose to answer or stop the interview at any time.

The interview will take between 30 minutes and one hour of your time and it will be audio recorded to ensure all verbal data is captured. The audiotape of your interview will be transcribed, and your identity will be anonymized. Interview notes or transcripts will be accessible only to researchers and analysts.

In recognition of your contribution, you will receive a $20 gift voucher at the completion of this telephone interview.

Are there any questions that you have now that I can answer for you?

**IF YES:** Answer participant’s questions and determine whether participant would be willing to take part.

Shall we start the interview?

**Interview guide for patients:**

| **Topic** | **Main questions** | **Prompting questions** |
| --- | --- | --- |
|  |  |  |
| **Experience with the EatSmart Program** | | |
|  | What was your experience with EatSmart program?  How useful did you find the EatSmart program? | -Not at all? …extremely useful?  -What were the MOST useful parts of the program and why did you find them useful?  -What the LEAST useful parts of the program were and why didn’t you find them useful? |
|  | Looking back at your experience with this program, what were 3 main messages that you remember? |  |
|  | Did you incorporate any of the Healthy eating tips into your daily life? | YES? Can you please explain what tip and how you have incorporated that into your daily life?  -No? can you please explain why? |
|  | After you completed the 3-month EatSmart program, over the past 6 months, have you changed the way you buy, cook or eat? (For example: -Did you eat more fruits and vegetable? Or high fiber products? Drink more water? Eat less unhealthy foods like chocolate, lollies or donuts…? Or -Did you buy more fruits and vegetables? Or -Did you add more vegetables to your dishes?) | -YES? Can you please explain how you have changed these and what helped you to do so?  What else might have helped you continue eating more fruits and vegetables after the program stopped?  -No? What was the primary barrier that prevented you from sticking to the lifestyle behaviours you learned?  -Straight after the program finished, in our survey in (Month), you mentioned that you had changed/hadn’t changed the way you buy, cook or eat …, can you tell us what happened after that and how is that going now? |
|  | (Future) How do you see your eating behaviour in the future? | Same as now? If yes, why? And If not what would you do differently? |
| **Views about the features of the website** | | |
| **Informational/educational content** | What are your views about the diabetes educational tips embedded in the website? I.e. the feature containing information on the importance of fruits and vegetables and totally healthy eating. | -Did you find them useful? -can you remember what Information was specifically useful for you?  -What was the least useful information in the website? Why, was it repetitive? Too simple? Too complicated?  - Is there any other information or topic that you would have liked to be included in the website? |
| **Videos, Recipes, Resources, quizzes** | Have you watched the **videos**? | Did you incorporate them in your daily life? What did you like the most about the videos? What did you like the least about the videos? |
|  | Have you checked the **recipes**? | -Did you try them in your daily life? -What did you like the most about the recipes? -What did you like the least about the recipes? |
|  | Have you read the **resources**? | Did you find them useful for you in your daily life? -What did you like the most about the resources? What did you like the least about the resources? |
|  | Have you checked the **quizzes**? | -What did you like the most about the quizzes? What did you like the least about the quizzes? |
| **Design (photos, colours, …)** | What do you think about the photos and visual messages in the website? | -Could they help you to understand the information more easily? -What did you like the most about them? -Was there anything you didn’t like about them or you would change? For example: did you like the website had more text than photos? |
| **Easy to use and navigate** | How did you find navigating the pages and finding information? | -Was it easy enough for you to go to Different modules? -Could you easily navigate through different parts of each module? - Was there anything you find particularly hard to use? - If you have problems with the website, what were they? What would you change about the design of the website to make it easier for users? |
| **Modules** | What do you think about the number of modules? Did you prefer more modules or there were enough? |  |
|  | What do you think about the length of each module? Did you prefer more information in each module or that was just enough? |  |
|  | What do you think about the time between introducing new modules? |  |
| **Text messages** | What do you think about the educational and reminder text messages which were sent to your phone 3 times a week? | Did they motivate you to visit website? Was there anything you dislike about these messages in particular? |
|  | What do you think about the number and frequency of text messages? Was is suitable for you? |  |
| **Final questions and Recommendations** | | |
|  | After you completed the 3-month EatSmart program, you did a survey for us in (Month) this year. We’re interested in knowing if you have used any of the program again since then! | -Yes? Which parts of the website did you look at?  -Were there benefits you got from visiting website in the last 6 months? If so, what were these?  -What factors in the EatSmart website could help you engage more with the program?  -No? What had stopped you from visiting the website again? For example: limited internet access, limited phone data? The content and information weren’t attracting? You have visited all parts in the first three months? Etc.! |
|  | Would you continue to visit the EatSmart website, if it were available? |  |
|  | Would you recommend EatSmart to other people with similar condition? |  |
|  | Was there anything you wanted or expected to see in the program but didn’t? |  |
|  | Is there anything we haven’t talked about that you think it is important to share before we end the interview? |  |

**“Thank you for the valuable information and your time.”**
